# The role of leadership level in college students’ facial emotion recognition: evidence from event-related potential analysis

**DOI:** 10.1186/s41235-023-00523-9

**Published:** 2023-12-20

**Authors:** Huang Gu, Shunshun Du, Peipei Jin, Chengming Wang, Hui He, Mingnan Zhao

**Affiliations:** 1 Department of Psychology, Faculty of Education, Henan University, Kaifeng, 475000 China; 2 Department of Psychology, School of Social and Behavioral Science, Nanjing University, Nanjing, 210023 China; 3 Department of Radiology, The Affiliated Drum Tower Hospital of Nanjing University, Nanjing, 210008 China; 4 School of Psychology, Center for Studies of Psychological Application, and Guangdong Key Laboratory of Mental Health and Cognitive Science, South China Normal University, Guangzhou, 510631 China; 5 Zhengzhou Polytechnic Vocational College, Zhengzhou, 451150 China; 6 Tianjin University of Sport, Tianjin, 301617 China

**Keywords:** Facial emotion recognition, Leadership, Event-related potentials, China, Time–frequency analysis

## Abstract

While the role of emotion in leadership practice is well-acknowledged, there is still a lack of clarity regarding the behavioral distinctions between individuals with varying levels of leadership and the underlying neurocognitive mechanisms at play. This study utilizes facial emotion recognition in conjunction with electroencephalograms to explore the temporal dynamics of facial emotion recognition processes among college students with high and low levels of leadership. The results showed no significant differences in the amplitude of P1 during the early stage of facial emotion recognition between the two groups. In the middle stage of facial emotion recognition, the main effect of group was significant on the N170 component, with higher N170 amplitude evoked in high-leadership students than low-leadership students. In the late stage of facial emotion recognition, low-leadership students evoked greater LPP amplitude in the temporal-parietal lobe when recognizing happy facial emotions compared to high-leadership students. In addition, time–frequency results revealed a difference in the alpha frequency band, with high-leadership students exhibiting lower alpha power than low-leadership students. The results suggest differences in the brain temporal courses of facial emotion recognition between students with different leadership levels, which are mainly manifested in the middle stage of structural encoding and the late stage of delicate emotional processing during facial emotion recognition.

## Introduction

Leadership has long been a central topic in the field of behavioral science. In recent decades, the study of youth leadership has also gained widespread attention. As future elites and potential leaders, college students need to develop strong leadership skills. In recent years, the concept of leadership has undergone a transformation. Traditional leadership theories, such as the trait theory, behavioral theory, and contingency theory, have been shown to have their limitations. Newer leadership theories, such as the charismatic leadership theory, transformational leadership theory, and value-driven leadership theory, have emerged and have gained prominence in the field of management.

### Leadership and emotion

Weber (1963) proposed the charismatic leadership theory, which emphasizes leaders’ unique personal charm and influence over followers. This theory acknowledges the malleability of leadership skills. Burns (1978) introduced transformational leadership theory, focusing on leader–follower interactions and inspiring followers through non-material means like appealing to emotions and developmental needs. Since the twenty-first century, value-driven leadership arouses followers’ cognitive schemas to enhance emotional connections and organizational identification (Bass & Riggio, 2006). Overall, emotions are playing an increasingly important role in leadership research, considered a crucial mechanism of leader influence and a conduit for information transfer during interactions.

Following these developments regarding the role of emotions in leadership, traditionally, cognitive factors were seen as the main predictors of leadership effectiveness. However, more recently, the role of emotions has gained prominence as an additional factor. Studies found emotional intelligence correlates positively with leadership effectiveness. For instance, Kerr et al. (2006) reported emotional intelligence predicted leadership effectiveness (*r* = 0.50) among corporate leaders. Also, Gómez-Leal et al.’s (2022) review revealed that emotional intelligence is key for effective leadership. Clearly, effective emotional abilities are critical for leadership, warranting further research.

Mayer and Salovey (1993) first proposed the concept of emotional intelligence as the ability to identify, monitor, and regulate emotions in self and others. The four key dimensions are: recognizing emotions, using emotions to facilitate thought, understanding emotions, and managing emotions. This model has also received some empirical support. In terms of emotion recognition, Rubin et al. (2005) found that leaders’ use of nonverbal cues to recognize emotions accounted for 20% of the variance in leadership effectiveness. Regarding emotion expression, Avolio et al. (2004) found that leaders’ appropriate emotional expression was positively correlated with leadership effectiveness. Trichas and Schyns (2012) found that when subordinates’ implicit leadership theories aligned with leaders’ facial emotions, subordinates had higher evaluative impressions of the leaders. For understanding and analyzing emotions, Toegel et al. (2013) argued that the process of understanding emotions is one of perspective-taking; leaders high in empathy are better able to accurately understand and meet subordinates’ needs, forming emotional bonds. Regarding using emotions to facilitate thinking, Visser et al.’s (2013) research found mediating effects of leaders’ emotions on the relationship between subordinates’ creativity and leadership effectiveness. For emotion regulation, Richards and Hackett (2012) found that when leaders and subordinates engaged in emotion regulation strategies, they were able to reduce experiences of failure and enhance leadership effectiveness, while excessive suppression of emotions decreased effectiveness.

### Facial emotion recognition

Through reviewing the concept of emotional intelligence, we can see that research on emotional intelligence involves multiple dimensions. In social environments, human faces are the most biologically significant visual stimuli. Faces contain a wealth of emotional cues, so the ability to recognize facial emotions is an important dimension of emotional intelligence. Whether one can accurately identify others’ emotional facial expressions, especially by gathering key social information from eyes, mouth, facial contours, etc., to infer inner states, emotions, and affective intentions, and respond accordingly, reflects to some extent the development of one’s social adaptability and overall competence (Orejarena et al., 2019). This ability relates to social interaction, job performance, and group survival and development. Scholars agree facial expressions are objective indicators of emotion. Individuals with high emotional intelligence can, on a personal level, better recognize, control, and regulate their own emotions. On an interpersonal level, they can more easily perceive others’ attitudes, emotions, and intentions. This has important significance for facilitating smooth social interaction and maintaining good interpersonal relationships. Recognizing others’ emotions has become a hot topic in psychology and cognitive neuroscience. A classic paradigm for studying facial emotion processing is the facial emotion recognition task (Montagne et al., 2007).

With increasing use of electrophysiological and neuroimaging techniques to study neural pathways for facial expressions, Luo et al., (2010) revised a three-stage temporal model of emotional facial processing: early direct visual processing, mid-level structural encoding, and late detailed affective analysis. The early stage involves rapid, automatic processing of low-level physical attributes, reflecting involuntary attention to stimuli. The mid-stage focuses on configural face processing, independent of feature information. The late stage involves further assessment of emotion-relevant details, differentiating faces by emotional valence. This model posits the visual processing, structural encoding, and expressive analysis stages are largely independent and occur in parallel. It has been widely applied in research on emotional facial recognition.

### ERP& facial emotion recognition

Event-related potentials (ERPs), also known as cognitive potentials or evoked potentials, refer to changes in electrical brain activity observed on the scalp surface using noninvasive brain electrical signal recording devices when specific stimuli or several types of stimuli act on the human sensory channels or a specific part of the brain. The neurophysiological changes of the brain reflect the changes in the cognitive function activity of the individual’s brain. Compared with functional magnetic resonance imaging, near-infrared and other technologies, ERP technology has the advantage of high time resolution at the millisecond level to record the process of brain cognitive processing activity. By extracting features from the collected brain electrical signals, emotionally processing-related feature indicators can be extracted. Previous studies on emotional face recognition have already divided the processing of faces into three stages of early, middle, and late and have also isolated the corresponding ERP components. In the early stages of emotional face recognition, there is a positive-going component P1 (mainly distributed in the occipital-temporal region, with a peak latency mainly between 80 and 130 ms after stimulus presentation) and a negative-going component N1 (widely distributed in various brain regions, with a peak latency in the prefrontal region between 60 and 140 ms after stimulus presentation). These early components are related to the individual’s attention to low-level physical features of the stimuli such as brightness and spatial resolution. P1 in emotional face recognition reflects the amygdala and occipital cortex’s crude but rapid monitoring of faces, while N1 reflects the individual’s attention capture by visual stimuli (Gu et al., 2019). In the middle stages of emotional face recognition, compared with non-face stimuli, face stimuli will evoke a negative-going component N170 (mainly distributed in the lateral temporal region and lower temporal region, with a peak latency mainly between 130 and 240 ms after stimulus presentation) in the posterior areas of the human brain. It typically peaks around 170 ms after stimulus presentation. Face stimuli can evoke a prominent N170 wave. Most studies consider N170 to be a face-specific component that is independent of attentional resources, not limited to emotion types, and has an automatic processing property, reflecting the structural encoding of face recognition (Hinojosa et al., 2015; Zhang et al., 2013). In the late stages of emotional face recognition, the P300 that appears near the central vertex and the subsequent positive slow wave, collectively referred to as the late positive potential (LPP), reflect higher-level processing such as conscious evaluation of emotional stimuli, working memory representation, decision making, and coping responses. They are not only influenced by attentional control but also related to the individual’s processing of emotional valence. Not only do emotional expressions elicit larger LPP amplitudes than neutral expressions, but negative expressions elicit larger LPP amplitudes than positive expressions (Luo et al., 2010).

Time–frequency analysis is a necessary complement to time domain signal analysis, which can observe and analyze components that are difficult to observe in the time domain signal. We can see the ERP waveform graph over time in a certain spatial state, and we can also see the increase or decrease of power values in different frequency bands to discuss the non-phase-locked neural oscillation information related to emotional processing by combining time domain and frequency domain information. Previous studies mainly analyze neural rhythmic oscillations in 5 frequency bands: Delta band (1–4 Hz), Theta band (4–8 Hz), Alpha band (8–13 Hz) and Beta band (14–30 Hz) as well as Gamma band (31–45 Hz) (Zhao, 2012). Alpha is the sleep wave, related to deep, subconscious and sleep states. The frequency of the Alpha band is generally defined in the range of 8–13 Hz, and different cortical regions of the brain have their own unique Alpha rhythms (such as motor cortex, visual cortex, etc.). Theta waves are related to intuition, creativity, recollection, imagination, depression, fatigue, intoxication states, and specific psychological tasks can also evoke significant Theta band oscillation activity. Oscillation in this band has a close relationship with working memory, and Theta band oscillation activity increases with the increase of memory load (Gu et al., 2020). Beta rhythms are modulated by different motor tasks and are associated with alertness, excitement, and highly concentrated task states. The Gamma oscillation rhythm is generally greater than 30 Hz and is mainly used in fields related to pain.

Above all, from the perspective of research on the relationship between leadership and emotion, although previous studies have made continuous improvements in measuring these constructs, ranging from single-structure dimensions to multi-structure dimensions and from ability models to mixed models, the traditional questionnaire-based measurements are limited in their ability to deeply explore the underlying neural mechanisms of the relationship between leadership and emotion. In our preliminary search of major databases, we found no studies specifically investigating the temporal course of leadership and emotion processing. Therefore, this study employed a questionnaire survey to assess leadership among college students in the surrounding area. Subsequently, based on the questionnaire results, participants with high and low leadership levels were selected and engaged in a subsequent facial emotion recognition task. The accuracy (ACC) and reaction time (RT) of participants in recognizing faces displaying three different emotional expressions were recorded, while their EEG signals were simultaneously collected using a 32-channel electroencephalogram system. The study aimed to analyze the differences between the two groups of participants in the temporal and time–frequency features during the emotion facial recognition task. Two hypotheses were proposed as follows: (1) College students with high and low leadership levels would exhibit behavioral differences in the facial emotion recognition task, with high-leadership students showing higher accuracy and shorter reaction times compared to low-leadership students. (2) College students with high and low leadership levels would demonstrate stage-specific differences in the EEG indicators during facial emotion recognition.

## Method

### Tools

Participants completed the self-report version of the Student Leadership Practices Inventory (SLPI: Kouzes & Posner, 2012), which was specifically designed for students. They assessed the frequency of their engagement in each of the 30 inventory items, which were rated on a five-point Likert scale ranging from 1 (rarely) to 5 (frequently). These items corresponded to five practices of exemplary leadership: (1) challenging the process; (2) inspiring a shared vision; (3) enabling others to act; (4) modeling the way; and (5) encouraging the heart. The total responses for each practice could range from 6 to 30, obtained by adding the response scores for each of the six behavioral statements related to that practice. To obtain the total score, the scores from the five dimensions of leadership practice were summed. The responses on the 30 items were also summed to create a composite scale for leadership, with a Cronbach’s alpha coefficient of internal reliability of 0.94 in the current study. The SLPI has demonstrated reasonably robust validity across multiple student populations (Posner, 2009).

### Participates

This study used G-power software to estimate the required sample size. With alpha = 0.05 for statistical significance in a two-tailed test, a sample size ≥ 530 was estimated to achieve high statistical power (1 − *β* = 0.95). Paper questionnaires were distributed to 760 university students from a certain university in Henan Province and surrounding universities, with 750 questionnaires returned. After excluding 19 invalid questionnaires (incomplete or all options were the same), 731 valid questionnaires remained, giving a valid return rate of 96.2%. The age of respondents was 17–25 years, with an average of 19.5 ± 1.14 years. Based on the SLPI (Posner, 2004) scores, the total leadership questionnaire scores were ranked and screened to select subjects in the top and bottom 5% for the high- and low-leadership groups. 35 subjects were selected for each group and contacted, but 7 students declined to participate in the experiment. Finally, 31 subjects were recruited for the high-leadership group, 32 for the low-leadership group, for a total of 63 subjects (30 males, 33 females; age range 17–23 years). Ultimately, participates completed a computerized version of a facial emotion recognition task while their EEG was recorded. All participants provided written informed consent before the experiment, and the assessment procedures were fully explained to them.

### Stimuli and procedures

**Fig. 1:**
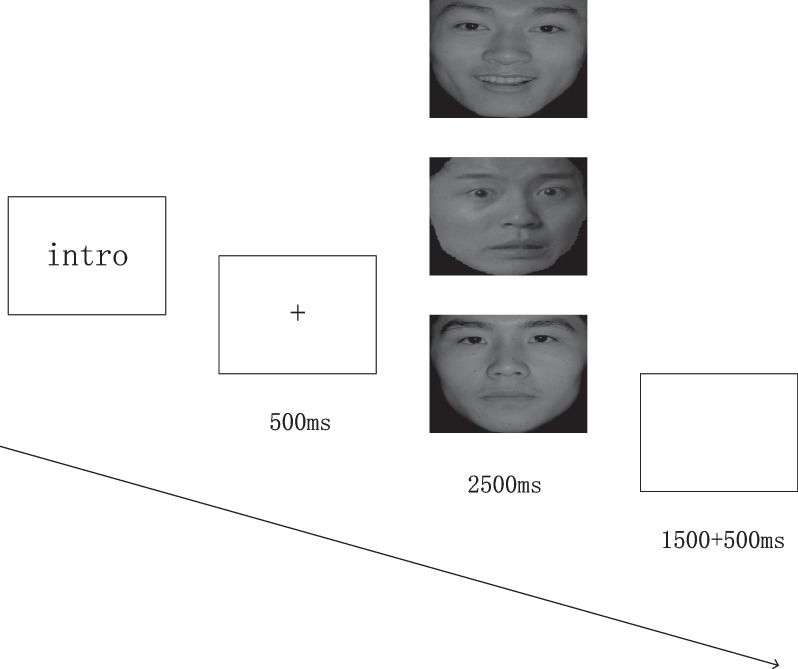
Procedures of the experimental tasks. Stimuli were composed of three emotions (happy, fear, and neutral)

The paradigm procedure was programmed using E-prime 2.0. We used the facial emotion recognition task experimental paradigm, which included stimulus images selected from the native Chinese Facial Affective Picture System (CFAPS) (Lu et al., 2005). The stimuli consisted of 40 fear emotion pictures, 40 happy emotion pictures, and 40 neutral emotion pictures, with an equal number of male and female faces in each category. Participants were seated in a tranquil room with their eyes approximately 100 cm from a 17-inch computer screen. They were instructed to distinguish happy, fear, and neutral emotions by pressing corresponding buttons (4 for right index finger, 5 for right middle finger, and 6 for right ring finger). The task consisted of 240 trials, each beginning with the presentation of a “ + ” for 500 ms. After the fixation, a stimulus was presented in the center of the screen (4.0° × 4.6° visual angle) for 2500 ms. This was followed by a blank screen lasting randomly from 1000 to 2000 ms before the next trial began. Prior to the actual task, a practice session consisting of 15 trials with randomly selected happy, fear, and neutral faces was conducted to ensure that participants fully understood the task (see Fig. 1).

### Statistic recording

To analyze the behavioral data, we conducted a repeated-measures analysis of variance (ANOVA) with ACC (proportion of correct responses) and RT (the interval of time between application of a stimulus and detection of a response) as dependent variables. Only correct responses were used for the RT analysis in this study. Emotion (happy, fear, neutral) was included as a within-subjects factor, and group (high leadership vs. low leadership) was included as a between-subjects factor. For effects with two or more degrees of freedom, we adjusted for violations of sphericity using the Greenhouse–Geisser correction.

### EEG recording

The EEG was recorded from a 32-channel scalp standard cap using the 10/20 system (Brain Products, Munich, Germany). We monitored the vertical electrooculogram (VEOG) by placing electrodes 1 cm from the outer canthi of the right eye and the horizontal electrooculogram (HEOG) by placing electrodes above and below the left eye. All electrode recordings were referenced online to FCz, and inter-electrode impedances were maintained below 5kΩ. The EEG and EOG signals were amplified using a 0.01–100 Hz band-pass filter and continuously sampled at 500 Hz/channel for offline analysis.

After data acquisition, EEG data were imported into the open-source MATLAB toolboxes EEGLAB and Letswave for neurophysiological data analysis. The EEG recordings were re-referenced to the average of the two mastoids and band-pass filtered between 0.1 and 30 Hz. Independent component analysis (ICA) was used to isolate ocular (blink and saccade) and other remaining artifacts. Epochs were extracted from 200 ms prior to the stimulus onset to 1000 ms post-stimulus interval, and baseline correction was performed using the mean voltage in the 200 ms interval preceding stimulus onset. ERPs were computed offline by averaging according to the experimental design. The data analyses were conducted using MATLAB R2013b (MathWorks, Natick, USA) and SPSS Statistics 20.0 (IBM, Somers, USA).

### ERP analysis

The present study focused on the potentials of three ERP components: P1, N170, and LPP. The selection of electrodes and time windows was based on previous relevant studies as well as visual inspection of the topographies in our study (Ji et al., 2021; Gu et al., 2019; Shim et al., 2016). Specifically, P1 amplitudes (130–200 ms) were analyzed at P3, P4, and Pz; N170 amplitudes (130–240 ms) were analyzed at P7 and P8; and LPP amplitudes (400–800 ms) were analyzed at C3, C4, Cz, P3, P4, and Pz. Consistent with previous studies, P1 and N170 amplitudes were measured as baseline-to-peak values, while LPP amplitude was measured as mean value.

### Time–frequency analysis

The present study analyzed the oscillatory power of the alpha frequency band (8–14 Hz) at O1 and O2 electrodes, using the continuous wavelet transform (CWT) on single-trial EEG epochs. Time–frequency representations were explored between 1 and 30 Hz in steps of 0.29 Hz, with epochs extracted from 200 ms pre-stimulus to 800 ms post-stimulus time points. To avoid edge effects when performing CWT, the pre-stimulus time interval (−400 ms to −200 ms) was used as a baseline interval. Based on average condition contrast maps and previous studies, a single cluster was tested: 8–14 Hz at 200–800 ms for alpha. The oscillatory power of this component was quantified as the mean amplitude within these time windows for each participant.

## Results

### Behavioral data

**Fig. 2:**
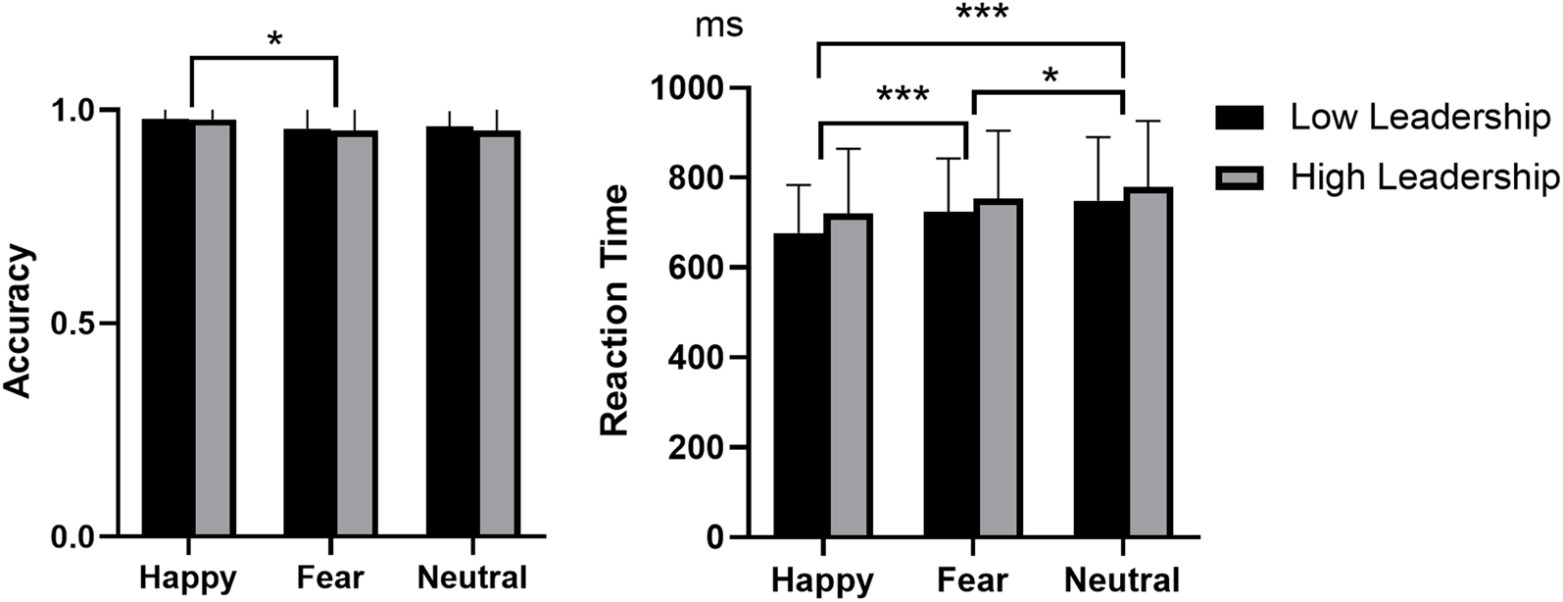
ACC and RT for three types of emotion between low-leadership and high-leadership groups

A repeated-measures ANOVA was performed for behavioral data analysis with group (high leadership vs. low leadership) as a between-subject factor and emotion (happy, fear and neutral) as a within-subject factor. Results showed a difference of emotion on accuracy [*F*(2, 122) = 3.426, *p* = 0.054,*η*^2^_p_ = 0.052], with happy emotion (*M* = 0.978, SD = 0.005) exhibiting higher accuracy than fear emotion (*M* = 0.954, SD = 0.013). But the difference between fear and neutral emotion (*M* = 0.955, SD = 0.008) was not found. The similar statistic measure was performed on reaction time, and we found the reaction time was significantly affected by emotion [*F*(2, 122) = 15.529, *p* < 0.001,*η*^2^_p_ = 0.203], with the reaction time in recognition of neutral emotion (*M* = 761.691, SD = 18.055) slower than fear emotion (*M* = 738.619, SD = 17.615) and the reaction time in recognition of fear emotion significantly slower than happy emotion (*M* = 695.989, SD = 16.312). (See Table 1 and Fig. 2).

**Table 1:** Mean accuracy and reaction time (Mean ± SD) of high-leadership vs. low-leadership undergraduates

	Happy	Fear	Neutral	F_EMOTION_ (*p*)	F_GROUP_ (*p*)	F_EMOTION*GROUP_ (*p*)
Accuracy						
Total	0.978 ± 0.005	0.954 ± 0.013	0.955 ± 0.008	3.426 (0.054)	0.196 (0.659)	0.059(0.882)
High leadership	0.980 ± 0.007	0.956 ± 0.019	0.960 ± 0.011			
Low leadership	0.977 ± 0.007	0.951 ± 0.017	0.951 ± 0.010			
Reaction time						
Total	695.989 ± 16.312	738.619 ± 17.615	761.691 ± 18.055	15.529(< 0.001***)	0.756 (0.388)	0.354 (0.681)
High leadership	676.362 ± 23.967	727.233 ± 25.880	751.198 ± 26.527			
Low leadership	715.615 ± 22.135	750.005 ± 23.902	772.185 ± 24.499			

*** p < 0.001

### ERP amplitude analysis

**Fig. 3:**
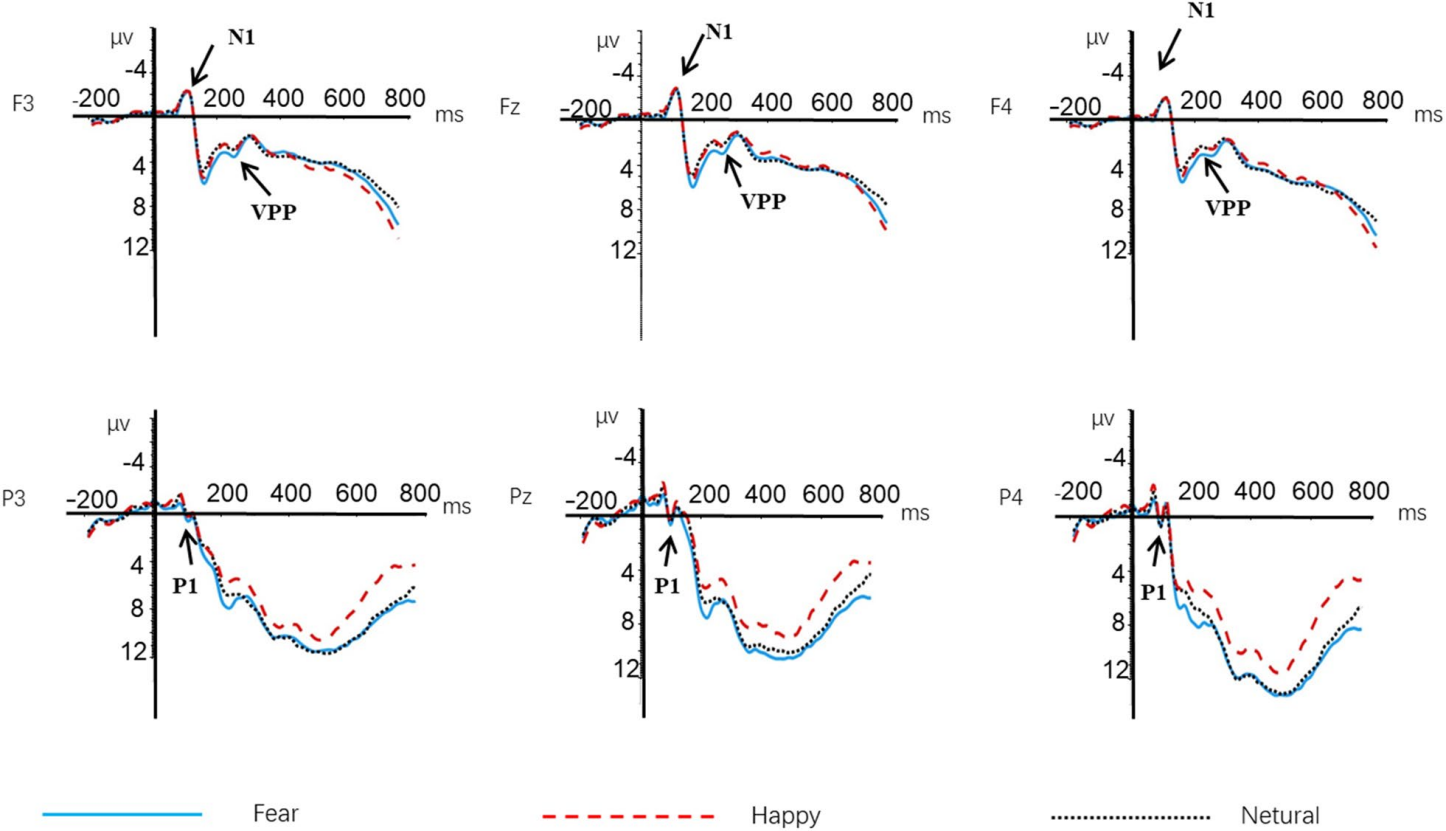
The amplitude of P1 elicited by faces with different emotions

For the consideration of space, we only included significant results in this part. Figure 3 shows the grand-averaged waveforms of P1, N170, and LPP between groups among three types of emotion.

#### P1

We conducted a repeated-measures analysis of variance on the maximum amplitude of the wave within the 130–200 ms time window following stimulus presentation, with emotion (happy, fearful, and neutral) and electrode (P3, P4, and Pz) as within-subject factors and group (high leadership and low leadership) as a between-subject factor. Results revealed a significant main effect of emotion [*F*(2, 122) = 4.373, *p* = 0.017, *η*^*2*^_*p*_ = 0.067], indicating that fearful emotion (*M* = 6.658, SD = 0.574) elicited larger P1 amplitudes than happy emotion (*M* = 5.551, SD = 0.547) and neutral emotion (*M* = 5.930, SD = 0.573). There was also a significant main effect of electrode [*F*(2, 122) = 20.585, *p* < 0.001, *η*^*2*^_*p*_ = 0.252), with Pz (*M* = 7.326, SD = 0.600) eliciting larger P1 amplitudes than P3 (*M* = 5.855, SD = 0.567) and P4 (*M* = 4.958, SD = 0.519). The main effect of group was not significant, [*F*(1, 61) = 2.148, *p* = 0.148, *η*^*2*^_*p*_ = 0.034]. The interaction between emotion and group was not significant [*F*(2, 122) = 0.903, *p* = 0.401, *η*^*2*^_*p*_ = 0.015]. The interaction between electrode and group was not significant [*F*(2, 122) = 1.267, *p* = 0.283, *η*^*2*^_*p*_ = 0.020]. The interaction between emotion and electrode was not significant [*F*(4, 244) = 0.643, *p* = 0.592, *η*^*2*^_*p*_ = 0.010]. The interaction among emotion, electrode, and group was not significant [*F*(4, 244) = 1.472, *p* = 0.223, *η*^*2*^_*p*_ = 0.024].

#### N170

A repeated-measures ANOVA was applied in this component with minimum amplitude between 130 and 240 ms as a dependent variable, emotion (happy, fear, and neutral), electrode (P7 and P8) as within-subject factors, and group (high leadership vs. low leadership) as a between-subject factor. Results showed a significant difference in group [*F*(1, 61) = 3.817, *p* = 0.05,*η*^2^_p_ = 0.059], with high-leadership group (*M* = − 1.940, SD = 0.569) elicited larger N170 amplitude than low-leadership group (M = − 0.355, SD = 0.578). The main effects of emotion were not significant [*F*(2, 122) = 1.111, *p* = 0.332, *η*^2^_p_ = 0.018]. Similarly, the main effects of electrode were not statistically significant [*F*(1, 61) = 2.576, *p* = 0.114, *η*^2^_p_ = 0.041]. The interaction between emotion and group also did not yield significant results [*F*(2, 122) = 0.783, *p* = 0.449, *η*^2^_p_ = 0.023]. Additionally, the interaction between electrode and group showed no significant effect [*F*(1, 61) = 2.836, *p* = 0.097, *η*^2^_p_ = 0.044]. The interaction between electrode and emotion was not significant [*F*(2, 122) = 0.009, *p* = 0.987, *η*^2^_p_ = 0.001]. Furthermore, the three-way interaction between electrode, emotion, and group was not significant [*F*(2, 122) = 0.487, *p* = 0.596, *η*^2^_p_ = 0.008].

#### LPP

**Fig. 4:**
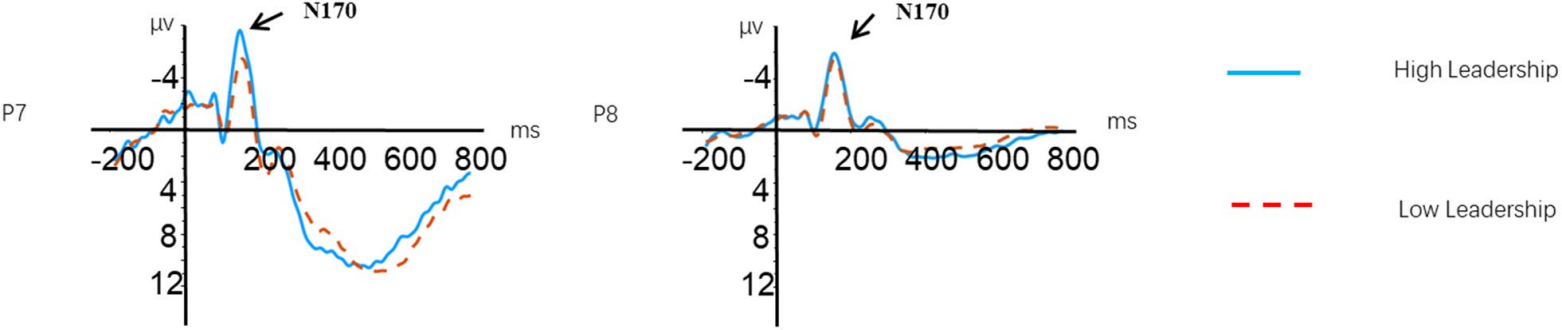
The N170 amplitudes for college students with different levels of leadership

**Fig. 5:**
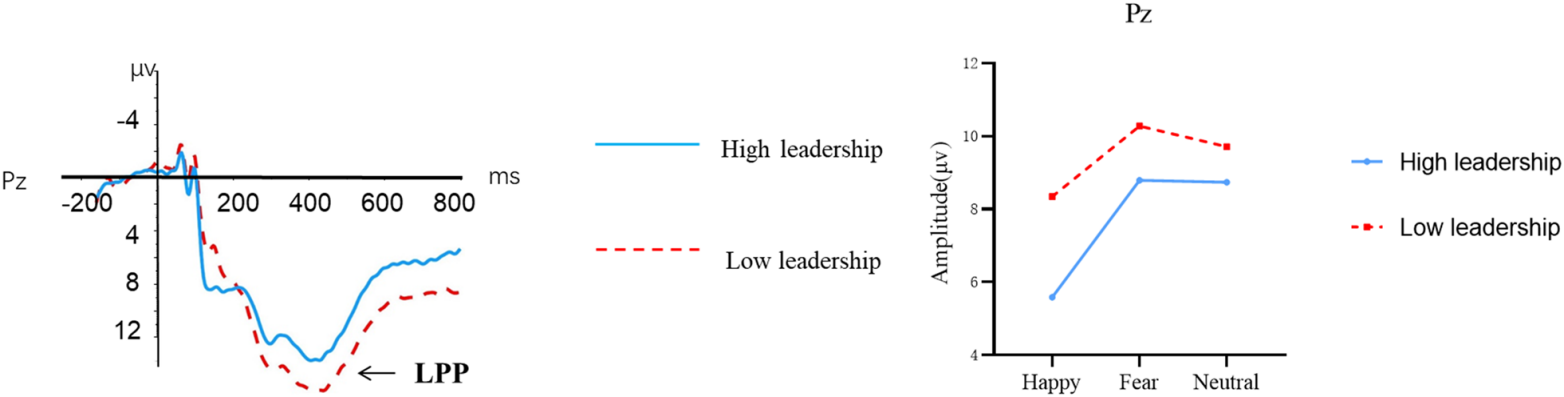
The LPP amplitude at the Pz electrode site under the interaction of group and emotion

A repeated-measures ANOVA was applied in this component with mean amplitude between 400 and 800 ms as a dependent variable, emotion (happy, fear, and neutral), electrode (C3, Cz, C4, P3, Pz, and P4) as within-subject factors, and group (high leadership vs. low leadership) as a between-subject factor. The results showed a marginally significant main effect of emotion on LPP amplitude [*F*(2, 122) = 2.806, *p* = 0.071, *η*^*2*^_*p*_ = 0.044], with fearful emotion (*M* = 8.884, SD = 0.805) eliciting greater LPP amplitudes than happy emotion (*M* = 7.177, SD = 0.953). The main effect of electrode was also significant [*F*(1, 61) = 11.061, *p* < 0.001, *η*^*2*^_*p*_ = 0.153], with frontal sites (i.e., C3, C4, Cz) (*M* = 8.738, SD = 0.815) showing larger LPP amplitudes than parietal sites (i.e., P3, P4, Pz) (*M* = 7.590, SD = 0.720), and midline sites (i.e., Cz, Pz) (*M* = 8.945, SD = 0.775) showing larger amplitudes than left hemisphere sites (i.e., C3, P3) (*M* = 7.773, SD = 0.767) and right hemisphere sites (i.e., C4, P4) (*M* = 7.775, SD = 0.732). The interaction between emotion × group × electrode was also significant [*F*(2, 122) = 3.362, *p* = 0.046, *η*^*2*^_*p*_ = 0.052], with lower-leadership college students (*M* = 10.282, SD = 1.274) showing greater LPP amplitudes compared to higher-leadership students (*M* = 8.423, SD = 1.294) when recognizing happy faces over parietal sites (see Figs. 3, 4 and 5).

### Time–frequency analysis

**Fig. 6:**
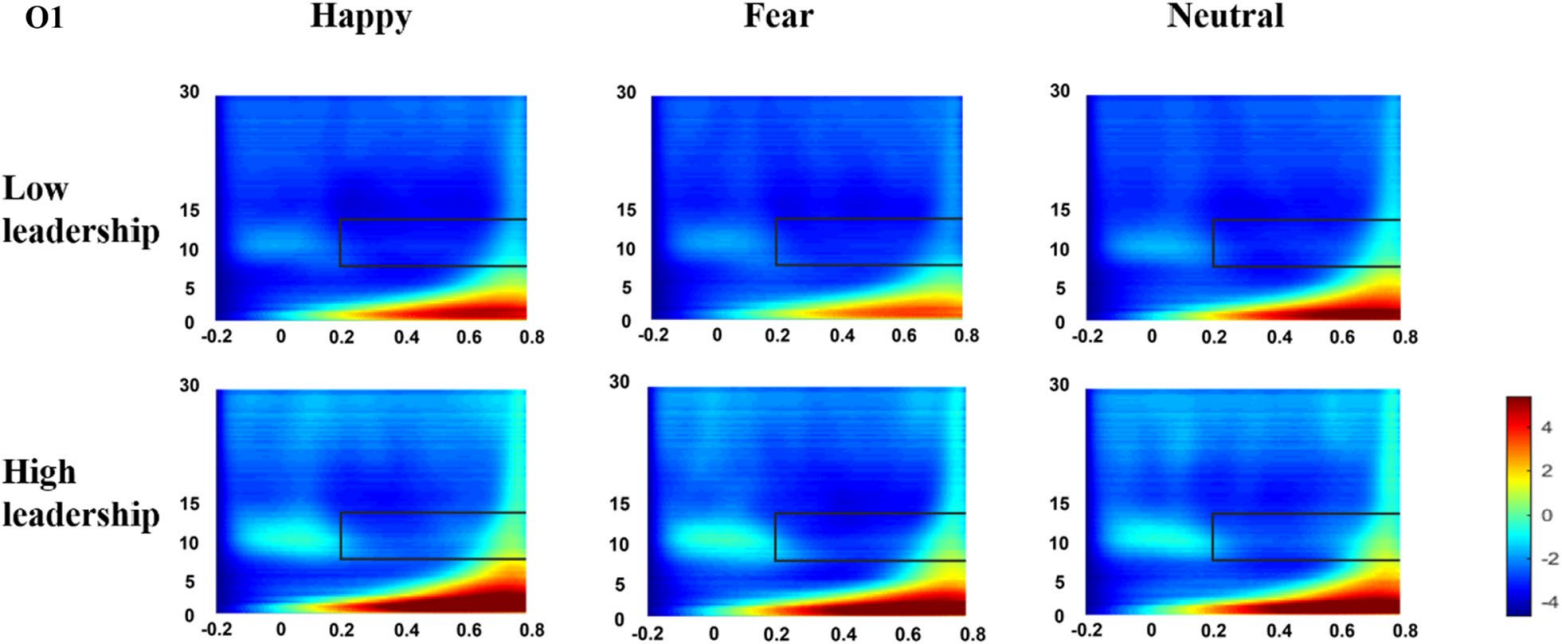
Group-averaged alpha band time–frequency spectrogram during facial emotion recognition task performance. Time (in ms) is denoted on the x-axis, with 0 ms defined as the onset of the stimuli. Frequency (in Hz) is shown on the y-axis

Time–frequency measures in alpha band showed a marginal significant difference in emotion [*F*(2, 122) = 2.935, *p* = 0.061, *η*^2^_p_ = 0.046], with fearful emotion (*M* = 1.753, SD = 0.160) eliciting enhanced alpha desynchronization compared to happy (*M* = 2.006, SD = 0.201) and neutral emotion (*M* = 2.016, SD = 0.200). There is a significant difference between groups [*F*(1, 61) = 4.745, *p* = 0.033, *η*^2^_p_ = 0.036], which revealed a reduced alpha desynchronization in low-leadership group (M = 2.305, SD = 0.236) compared to high-leadership group(*M* = 1.546, SD = 0.256). The main effect of electrode was not significant [*F*(1, 61) = 0.182, *p* = 0.671, *η*^2^_p_ = 0.003]. The interaction between emotion and group was not statistically significant [*F*(2, 122) = 0.711, *p* = 0.483, *η*^2^_p_ = 0.012]. The interaction between electrode and group also failed to yield statistical significance [*F*(1, 61) = 0.478, *p* = 0.492, *η*^2^_p_ = 0.008]. The three-way interaction among emotion, electrode, and group was likewise non-significant [*F*(2, 122) = 1.193, *p* = 0.306, *η*^2^_p_ = 0.019]. (See Fig. 6).

## Discussion

The present study aimed to investigate the neural underpinnings of emotional face processing in high-leadership and low-leadership college students. The results indicated no significant differences between the two groups in terms of accuracy and reaction time. However, several significant findings were observed from the ERP data analysis.

### Early stage of emotion face recognition

In the early stage of emotion face recognition, this study focused on exploring the role of the early components P1 elicited from the frontal-parietal lobes in emotion face recognition between the two groups. The results showed that the main effect of emotional face on P1 amplitude was significant, with fearful faces eliciting greater P1 amplitudes than happy and neutral faces. This indicates that visual processing resources are automatically captured by negative emotional faces during pre-attentive processing before automation in college students, which may imply that individuals need more attentional resources to process the threatening information conveyed by negative emotional faces (Jiang et al., 2014). The face processing model proposed by Adolphs (2002) suggests that there is a fast perceptual stage jointly executed by brain regions including the striate cortex, subcortical tissues, and amygdala in the early stage of face processing. The finding of the P1 component verifies the validity of this model. In the emotion face recognition task, the P1 component reflects the coarse but rapid categorization of facial information conducted by the early detection system composed of the above brain regions (Schindler et al., 2021). Fearful faces convey threatening or hostile information that is vital to individuals’ adaptability, while happy faces convey friendly information that is more common in interpersonal interactions, and neutral faces convey uncertain information. Our study found that participants showed greater brain responses to negative emotional faces, consistent with Luo et al., and and’s (2010) finding that fearful faces led to enhanced P1 amplitudes in the early stage of emotion face recognition. In addition, the main effect of group on P1 amplitude was not significant. Previous studies have used the P1 component to examine individuals’ proximal attention to salient physical features of space and objects (Smith et al., 2003). In the field of emotion face recognition, P1 also reflects the rapid extraction and unconscious processing of emotional information (Vuilleumier & Pourtois, 2007). The lack of significant differences in P1 amplitudes between the two groups indicates no obvious differences between high- and low-leadership college students in the early stage of emotion processing.

### Middle stage of emotion face recognition

In the middle stage of emotion face recognition, one of our main findings is that the main effect of group on N170 amplitude was significant, with higher-leadership college students eliciting greater N170 amplitudes than lower-leadership students. Brain magnetic and electrical studies have shown that a negative-going component N170 occurring around 170 ms after stimulus onset can be observed near the temporal-parietal region, reflecting the encoding process of facial configuration according to the cognitive economy principle. In addition, N170 also reflects individuals’ visual experience (familiarity level). Some studies have found that when individuals learn something new and become skilled to a certain level, larger N170 responses can also be elicited. For example, Rossion et al. (2002) found that native language could elicit N170 components similar to faces. Herrmann (2007) found same-race faces elicited larger N170 than other-race faces. Larger N170 indicates greater neural activity in encoding facial configuration, suggesting greater brain flexibility in processing facial structural information and representing better facial configuration encoding ability (Yang et al., 2020). Since emotion face recognition paradigms have been mostly conducted in special participant groups, the smaller N170 amplitudes elicited by patients with severe depression, ADHD, and schizophrenia compared to normal controls also indirectly concur with our experimental results (Aydin et al., 2023; Chen et al., 2022; Salisbury et al., 2019).

In addition, we also found that the main effect of emotion and other interaction effects did not significantly affect N170 amplitude. This indicates that N170 is a face configuration specific component, unaffected by feature information like emotional valence. First, according to Bruce and Young's emotion face processing stage model, facial configuration encoding and expression processing proceed independently, with facial configuration encoding unaffected by emotional information. Thus, no interaction exists between N170 and emotional valence (Bruce & Young, 1986). Our lack of finding an effect of emotional valence on N170 amplitude also supports this model. Krumhuber et al. (2019) manipulated the orientation of emotional faces and their valence and found N170 unaffected by face orientation or valence, verifying Bruce’s hypothetical model. Subsequent studies using 6 basic emotions also obtained consistent conclusions.

### Late stage of emotion face recognition

In the late stage of emotion processing, first, we found a marginally significant main effect of emotional face on LPP amplitude, with fearful faces eliciting larger LPP than happy and neutral faces. LPP refers to the late positive component elicited from the central-parietal region 300 ms after emotion face presentation. During emotion processing, LPP reflects the conscious processing of emotional stimuli by the cerebral cortex. In this process, the brain evaluates the attributes of stimuli, represents memories, and makes decisions and responses based on task requirements (Schupp et al., 2006). Frühholz et al. (2009) examined ERP components elicited when background and face colors were consistent or inconsistent. They found larger LPP amplitudes in the inconsistent than consistent condition, with negative emotional faces eliciting larger LPP than other valences. Hietanen and Astikainen (2013) compared ERP components for emotional faces and pictures and found larger LPP for faces, especially for negative emotions. Luo et al. (2010) used Chinese emotional faces and Chinese participants, proposing a three-stage model of emotion processing, with negative faces eliciting larger LPP than positive or neutral faces in the late stage. Second, we found lower-leadership college students showed larger LPP amplitudes than higher-leadership students for happy faces, suggesting slower attentional responses to positive stimuli for lower-leadership individuals, implying some insensitivity to positive emotional stimuli. Previous studies on negative emotion processing biases in anxiety disorders, post-traumatic stress disorder (PTSD), and various phobias also found larger LPP amplitudes in patients than healthy controls.

### Neural oscillation characteristics

In addition to ERP analysis, this study also used continuous wavelet transform to examine the dynamic neural oscillation information elicited during emotion face recognition between the two groups. Similar to the ERP results, the main effect of group was significant, with lower Alpha power for the higher-leadership group. Alpha power is an important indicator of individuals’ attention, memory, and agility. When individuals are awake, the magnitude of Alpha waves is negatively correlated with cortical activity in corresponding brain regions. That is, when concentrating on a stimulus, Alpha power decreases, while not focusing on a stimulus increases Alpha power (Keitel et al., 2019). Keune et al.’s (2017) study on the relationship between resting state neural oscillations and cognitive function in multiple sclerosis patients found Alpha power increases negatively correlated with processing speed. A magnetoencephalography (MEG) study revealed a positive predictive relationship between Alpha power and cognitive decline (Schoonheim et al., 2013). Our results show greater Alpha desynchronization associated with reduced brain activity in the lower-leadership group, suggesting differences in attention, memory and other cognitive processes during emotion face recognition compared to the higher-leadership group.

However, our study has some limitations that need to be addressed. Firstly, our sample only included college students. It is unclear whether these findings can be generalized to managers in business and public-sector organizations, which operate in significantly different environments. Future studies should aim to replicate our findings in a more diverse sample. Secondly, the measurement of leadership in our study was self-reported by the participants, which may have been influenced by social desirability bias. Future studies could incorporate additional measures of leadership, such as evaluations from teachers, classmates, or other objective sources. This would allow for a more comprehensive assessment of the participants’ leadership abilities.

## Conclusion

In summary, this study found that high leadership level college students exhibited larger N170 amplitudes, while low-leadership group students exhibited larger LPP amplitudes under an interaction of emotion valence, as analyzed using the ERPs P1, N170, and LPP. Additionally, our results showed attenuated brain oscillations (alpha) in low-leadership college students. These findings suggest that deficits in facial emotion processing, particularly among low-leadership individuals, may be due to impairments in visual processing, facial structure encoding, attention, and emotional semantic distribution abilities.

## Data Availability

The data and materials supporting the findings of this study are available from the corresponding author upon reasonable request.

## Abbreviations

EEG: Electroencephalography
ERPs: Event-related potentials
LPP: Late positive potential
ACC: Accuracy
RT: Reaction time
SLPI: Student leadership practices inventory
CFAPS: Chinese facial affective picture system
ANOVA: Analysis of variance
VEOG: Vertical electrooculogram
HEOG: Horizontal electrooculogram
ICA: Independent component analysis
CWT: Continuous wavelet transform
PTSD: Post-traumatic stress disorder
MEG: Magnetoencephalography
