# Correction: The role of leadership level in college students’ facial emotion recognition: evidence from event-related potential analysis

**DOI:** 10.1186/s41235-024-00536-y

**Published:** 2024-02-16

**Authors:** Huang Gu, Shunshun Du, Peipei Jin, Chengming Wang, Hui He, Mingnan Zhao

**Affiliations:** 1 Department of Psychology, Faculty of Education, Henan University, Kaifeng, 475000 China; 2 Department of Psychology, School of Social and Behavioral Science, Nanjing University, Nanjing, 210023 China; 3 Department of Radiology, The Affiliated Drum Tower Hospital of Nanjing University, Nanjing, 210008 China; 4 School of Psychology, Center for Studies of Psychological Application, and Guangdong Key Laboratory of Mental Health and Cognitive Science, South China Normal University, Guangzhou, 510631 China; 5 Zhengzhou Institute of Technology, Zhengzhou, 450000 China; 6 Tianjin University of Sport, Tianjin, 301617 China

**Correction:Cognitive Research: Principles and Implications (2023) 8:73** 10.1186/s41235-023-00523-9

The original article [1] contains an error in affiliation #5. The corrected affiliation information can be viewed in the affiliation information of this article and ahead:

5. Zhengzhou Institute of Technology, 450000, China

## References

[1] Gu H (2023). The role of leadership level in college students’ facial emotion recognition: evidence from event-related potential analysis. Cognitive Research: Principles and Implications.

